# Yokukansan (TJ-54) for treatment of pervasive developmental disorder not otherwise specified and Asperger’s disorder: a 12-week prospective, open-label study

**DOI:** 10.1186/1471-244X-12-215

**Published:** 2012-11-29

**Authors:** Tsuyoshi Miyaoka, Rei Wake, Motohide Furuya, Kristian Liaury, Masa Ieda, Kazunori Kawakami, Keiko Tsuchie, Takuji Inagaki, Jun Horiguchi

**Affiliations:** 1 Department of Psychiatry, Shimane University School of Medicine, 89-1 Enyacho, Izumo, 693-8501, Japan

**Keywords:** Pervasive developmental disorder not otherwise specified, Asperger’s disorder, Irritability, Yokukansan (TJ-54)

## Abstract

**Background:**

Numerous medications have been tested on patients with pervasive developmental disorder not otherwise specified (PDD-NOS) and Asperger’s disorder. Although many of these medications have been demonstrated to be useful, no clear primary treatment for PDD-NOS and Asperger’s disorder has emerged. Despite the efficacy of some of the medicines, the acceptability and side effects have proven to be barriers to their use. Recent studies indicate that the traditional Japanese herbal medicine yokukansan (TJ-54) may be safe and useful in treating behavioral and psychological symptoms in dementia and some neuropsychiatric disorders. We aimed at evaluating both the efficacy and safety of TJ-54 in patients with well-defined PDD-NOS and Asperger’s disorder.

**Methods:**

This was a 12-week prospective, open-label investigation of TJ-54 in 40 children, adolescents, and adults diagnosed with PDD-NOS or Asperger’s disorder. Primary outcome measures included the Clinical Global Impressions-Severity of Illness Scale (CGI-S) and the Aberrant Behavior Checklist-Iritability subscale score (ABC-I).

**Results:**

Forty subjects, ages 8–40 years (mean 22.7 ± 7.3 years) received a mean final TJ-54 dosage of 6.4 ± 1.3 g/day (range 2.5-7.5 g/day). Full-scale intelligence quotient (IQ) scores ranged from 70 to 110 (mean 88.9 ± 13.2). Thirty-six (90%) of 40 subjects showed fewer interfering symptoms of irritability, including aggression, self-injury, and tantrums, with a final CGI-S of 1 or 2 (normal, not at all ill or borderline mentally ill) and a 80% or greater improvement on the ABC-I. The mean CGI-S score at baseline was 6.8 ± 0.8 whereas scores at end point was 1.9 ± 0.1 (< 0.0001). ABC-I scores ranged from 11 to 29 (mean 17.4 ± 3.66) at baseline, whereas scores at week 12 ranged from 0 to 5 (mean 0.93 ± 0.97) (p <0.0001). TJ-54 was well tolerated. No subject exited the study due to a drug-related adverse event.

**Conclusions:**

These preliminary data suggest that TJ-54 may be effective and well tolerated for treatment of severe irritability, lethargy/withdrawal, stereotypic behavior, hyperactivity/noncompliance, and inappropriate speech in patients with PDD-NOS or Asperger’s disorder. However, given the characteristics of this trial, the present findings should be taken cautiously, and larger-scale placebo-controlled studies are needed to elucidate the efficacy and tolerability of TJ-54 in this understudied population.

## Background

Pervasive developmental disorder not otherwise specified (PDD-NOS) and Asperger’s disorder are understudied neuropsychiatric disorders characterized by core social and communication impairments and repetitive interests and activities [1]. Moreover, severe irritability, aggression, self-injury, and tantrums are observed frequently and can have a significant impact on the affected individual and his or her family [2,3]. Despite numerous treatment studies of autistic disorder (autism), research focused specifically on the pharmacotherapy of PDD-NOS and Asperger’s disorder is greatly lacking [4]. Recent epidemiological surveys of PDDs suggest that their prevalence may be increasing, with conservative estimates of autism at 63.7/10,000, Asperger’s disorder at 6/10,000, and PDD-NOS at 37.1/10,000 people in the worldwide [5,6]. These findings have heightened awareness of the various subtypes of PDDs.

The evidence-based practice guideline for the treatment of patients with PDD-NOS and Asperger’s disorder stresses the role of behavior interventions or psychosocial treatment as the first-line treatment [4], in combination with symptom-targeted adjunctive pharmacotherapy [7]. Pharmacotherapy has a relevant role in treating both state symptoms during periods of acute decompesation and trait vulnerabilities [8]. Different PDD-NOS and Asperger’s disorder symptom clusters may be responsive to a range of pharmacological agents. Since the introduction of atypical antipsychotic drugs, rates of ‘off label’ use for the treatment of PDD-NOS and Asperger’s disorder have increased [9-15]. Serotonin reuptake inhibitors and mood stabilizer are of limited efficacy in patients with PDD-NOS and Asperger’s disorder [16].

Yokukansan (TJ-54) was developed in 1555 by Xue Kai as a remedy for restlessness and agitation in children [17]. Prompted by the increasing life expectancy of the Japanese population, geriatricians have begun to use this traditional regimen for behavioral and psychological symptoms of dementia (BPSD) in the elderly. For example, Iwasaki et al. reported two cases of BPSD in an extended care unit that were successfully treated with TJ-54 [18,19]. Iwasaki et al. reported that TJ-54 improved BPSD and activities of daily living in a randomized, observer-blind, control trial [20]. In this study subject number is fifty-two patients with mild-to-severe dementia (Alzheimer’s disease, 30; vascular dementia, 9; Alzheimer’s disease with CVD; dementia with Lewy bodies, 10). We previously reported that TJ-54 therapy is a well-tolerated and effective remedy that ameliorates the symptoms of several neuropsychiatric disorders (borderline personality disorder, drug-induced tardive dyskinesia, schizophrenia, and visual hallucination due to vision loss) [21-26]. Such detailed case observations and clinical trials of this traditional Japanese medicine studied, can suggest new treatments for PDD-NOS and Asperger’s disorder. TJ-54 extract is a kampo medicine containing JP Atractylodes lancea rhizome, JP poria sclerotium, JP Cndium rhizome, JP Uncaria hook, JP Japanese angelia root, JP Bupleurum root, and JP Glycyrrhiza (JP: Japanese Pharmcopoeia). TJ-54 has been used for the treatment of insomnia, irritability and convulsion for many years, and the pharmacological effects of TJ-54 is thought to be associated with 5-HT receptors [19,20].

This study aimed to evaluate the efficacy and tolerability of TJ-54 in patients with PDD-NOS and Asperger’s disorder. Our hypothesis was that TJ-54 would reduce some symptoms of PDD-NOS and Asperger’s disorder, resulting in improved social and global functioning. This study should be considered a pilot study permitting the development of a future randomized comparative study.

## Methods

### Patient population

Subjects were recruited after a preliminary psychiatric interview and further assessment at the Outpatient Community Mental Health Unit of Shimane University School of Medicine, Izumo, Japan. Subject inclusion criteria were as follows: age > 7 years; diagnosis of PDD-NOS and Asperger’s disorder according to a structured clinical interview (SCID I, clinician version) [27] for DSM-IV criteria; and absence of axis I comorbidity, as informed by SCID II [28]. Although the score of hyperactivity are high, in this study, the patients with ADHD were not included. Patients were excluded if they were suffering from any major medical or neurological illness, or pregnant, lactating, or of child-bearing age and not using adequate contraceptive precautions. Demographic and clinical characteristics of the sample are listed in Table 1. This study approved by the Helsinki Committee (institutional review board) of the Department of Psychiatry of the Shimane University School of Medicine. All patients/guardians provided written informed consent to participate, and subjects provided written informed assent when possible, according to institutional guidelines and the recommendations in the Declaration of Helsinki.

**Table 1:** Demographic and clinical characteristics of 20 subjects diagnosed with BPD completing a 12-week trial with YGS

**Sociodemographic and clinical variables**	**Value**
Sex, N (male/female)	22/18
Age, mean±SD, years	22.7 ± 7.3
IQ	88.9 ± 7.3

### Participants

Forty children, adolescents, and adults 8–40 years were enrolled. Diagnoses of PDD-NOS or Asperger’s disorder using Diagnostic and Statistical Manual of Mental Disorders, 4^th^ edition, Text Revision (DSM-IV-TR) [1] criteria were made by a board-certified child and adolescent psychiatrist experienced in the assessment and diagnosis of PDDs. Subjects were required to have a mental age of at least 18 months, as determined by the Wechsler Intelligence Scale [29], and to be physically healthy. Additional inclusion criteria included a CGI-Severity (CGI-S) scale [30] score of at least 4 (“moderately iII”) focused specifically on target symptoms of irritability (aggression, self-injury, tantrums) and a score >18 on the ABC [31,32]. Physicians (T.M., T.I., R.W., M. F.) filled out the ABC scale and CGI-S scale. Subjects with a DSM-IV-TR diagnosis of another PDD, other primary psychiatric disorder, active seizure disorder, significant medical condition, or positive urine pregnancy test were excluded.

### Study design

A 12-week prospective, open-label study design was chosen to gather pilot data on the effects of TJ-54 in children, adolescents, and adults with PDD-NOS or Asperger’s disorder in anticipation of larger-scale, double-blind, placebo-controlled studies of this population. The study duration allowed for gradual titration as well as a 4-week maintenance phase. All subjects underwent a screening and baseline visit. Follow-up visits occurred every two weeks during the 12-week open-label trial period.

### Dosage

All subjects initially received 2.5 g/day of TJ-54 for 3 days. The dosage was then increased to 5.0 g/day and continued to the end of week 2. The investigators then increased the dosage to maximum of 7.5 g/day over the next 4 weeks if optimal clinical response had not occurred and intolerable adverse effects had not emerged. The dosage maintenance phase lasted 8 weeks at the optimal dosage.

Nonpharmacologic therapy (e.g., psychotherapy and behavior modification) was permitted provided, it was stable before screening and consistent throughout the study.

### Primary outcome measures

The primary outcome measures were the CGI-S and ABC-I. The CGI-S is a scale designed to assess global change from baseline. In this study, the rater scored the CGI-S with regard to severity of irritability, including aggression, self-injury, and tantrums. The CGI-S rates subjects from 1 to 7 (1 = normal, not at all ill; 2 = borderline ill; 3 = mildly ill; 4 = moderately ill; 5 = markedly ill, 6 = severely ill; and 7 = among the most extremely ill patients). The ABC was used as a primary outcome measure in the aforementioned studies by the RUPP Autism Network [33,34] and Shea and colleagues [35]. The ABC is a 58-item checklist that measures six areas of behavior: irritability, lethargy/withdrawal, stereotyped behavior, hyperactivity and inappropriate speech [31,36] and gives a total composite that has confirmed reliability and validity in regard to the factor structure, distribution of scores, and sensitivity to change. The Irritability subscale consists of 15 items on temper tantrums, aggression, mood swings, irritability, property destruction, and self-injury. The CGI-S and ABC were administered at every visit after baseline. Patients were rated between 10 AM and noon at baseline and after 2, 4, 6, 8, 10, and 12 weeks. A videotape of the subject was obtained, with and without family in place whenever applicable at clinic, and 3 standardized clips lasting 15 min were generated while the subject was rest, talking , and listening. These clips were subsequently proposed in random order to four psychiatrist (T.M., T. I., R.W., and M.F.) with experience in CGI-S and ABC-I assessment, unaware of the study protocol, blind to the treatment, who rated all clips in random order, and the scores for each subject and session were then averaged. The intraclass correlation coefficient [37] for total scores, based on 12 randomly selected ratings, was for 0.81 (P < 0.001) for the ABC-I and 0.83 for the CGI.

### Safety assessment and monitoring

Medical and disease history, physical examination, body weight, blood pressure, and electrocardiograms were assessed at baseline. Laboratory studies included a baseline screening for liver disease, metabolic dysfunction (ie, glycemia, cholesterol, triglycerides), kidney disease, electrolyte imbalance, anemia, adequate blood cell and platelet counts, and prolactin levels. At each visit, a medical-psychiatric-neurological review was carried out, together an assessment of body weight and a review of adverse events and concomitant medications. The above laboratory studies were reassessed at week 12. Treatment-emergent adverse events were elicited at each bi-weekly visit by the treating clinician using the Safety Monitoring Uniform Report Form, a semistructured review of body systems [38].

### Statistical analysis

All data were expressed as means ± SD. A paired *t* test was used to compare the differences between the means of CGI-S and ABC-I scores before and after treatment. The Fisher exact test was used to examine the difference in the proportion of side effects before and after treatment. The statistically significant difference was set a P < 0.05. Statistical analysis of data was carried out using SPSS Text Analysis for Surveys 4.0.1.

## Results

Of 50 subjects screened, 40 (80%) met eligibility criteria and were enrolled. All participants were Japanese. The sample consisted of 22 males and 18 females, aged 11–35 years (mean 22.7 ± 7.3 years). Twenty-one subjects were diagnosed with PDD-NOS, and ninety-one subjects were diagnosed with Asperger’s disorder. Full-scale intelligence quotient (IQ) scores ranged from 70 to 110, with a mean score of 88.9 ± 13.2. Subjects received a mean final TJ-54 dosage of 6.4 ± 1.3 mg/day (2.5-7.5 g/day). All subjects completed the study.

### Treatment response

**Figure 1:**
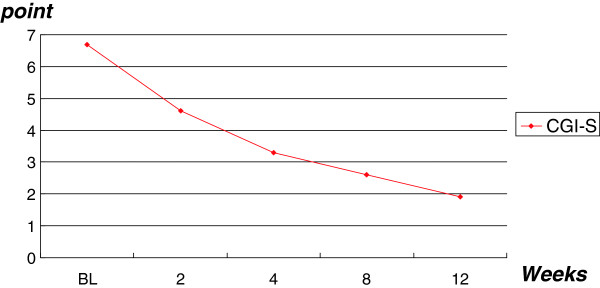
**Changes in mean CGI-S scores.** CGI, Clinical Impressions-Severity; BL, baseline.

**Figure 2:**
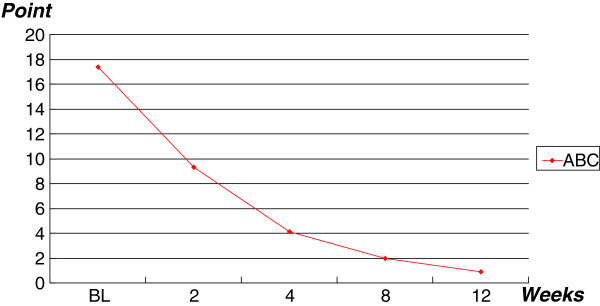
**Changes in mean ABC scores. **ABC, Aberrant Behavior checklist.

Thirty-six (90%) of 40 subjects were considered responders, as determined by a CGI-S score of 1 or 2 and a > 25% improvement on the ABC-I. All 19 subjects diagnosed with Asperger’s disorder responded to treatment, whereas 17 of 21 (81%) subjects with PDD-NOS responded. The mean CGI-S score at baseline was 6.8 ± 0.8 whereas scores at end point was 1.9 ± 0.1, with 36 (90%) of 40 subjects rated as much or very much improved in regards to interfering target symptoms of irritability (aggression, self-injury, tantrums) (p < 0.0001) (Figure 1 and Table 2). ABC-I scores ranged from 11 to 29 (mean score 17.4 ± 3.66) at baseline, whereas scores at week 12 ranged from 0 to 5 (mean score, 1.95 ± 0.7) (p < 0.001) (Figure 2 and Table 2).

**Table 2:** Baseline score and group change in behavior after treatment with TJ-54 for 12 weeks

		**Baseline Group**		**12-week Group**		**Group Mean**		**p1**
		**Baseline Group**		**12-week Group**		**Group Mean**		**p1**
Measure		Mean	SD	Mean	SD	Mean	SD	
CGI-S		6.78	0.83	1.95	0.11	-4.825	0.46	<.0001
ABC-I	(Irritability)	17.4	3.66	0.925	0.971	-16.5	1.46	<.0001
ABC-Total		77.8	11.4	6.90	4.84	-70.9	5.21	<.0001
	Lethargy	23.7	3.68	2.10	1.87	-21.6	1.78	<.0001
	Stereotypy	9.23	3.78	1.33	1.37	-7.90	1.55	<.0001
	Hyperactivity	21,7	3.90	1.83	1.89	-19.9	1.67	<.0001
	Inappropriate speech	5.78	1.25	0.73	0.75	-5.05	0.631	<.0001

1 Significance by t test.

2 Negative values indicate a positive change.

### Safety measures and adverse effects

Laboratory parameters were within the normal range at baseline and remained in the reference range for the whole sample throughout the 12-week trial. There were no reports of serious adverse effects attributable to the study drug. The adverse effects were mild and transient in five cases (nausea). TJ-54 was well tolerated overall, with no severe or serious adverse effects recorded during the study. None of the adverse effects was treatment limiting. No subjects exited the study due to a drug-related adverse event.

## Discussion

The results of this 12-week prospective, open-label study suggest that TJ-54 may be effective and well tolerated for children, adolescents and adults with PDD-NOS and Asperger’s disorder. Although the drug may also be effective in patients with PDD-NOS and Asperger’s disorder, our small sample size limits interpretation of data in these PDD subtypes.

In order to clarify the mechanism underlying the amelioration of aggressiveness by TJ-54, the effects of this medicine on the aggressive behaviour observed in rats treated with a serotonegic neurotoxin, para-chloroamphetamine, were investigated. TJ-54 ameliorated the aggressive behavior as effectively as a serotonin (5-HT) 1A receptor agonist or 5-HT2A receptor antagonist [39]. From these results, we hypothesized that this effect of TJ-54 may be due to 5-HT1A receptor antagonism [40].

An in vitro binding study demonstrated that TJ-54 showed agonistic binding to 5-HT1A and dopamine (DA) 2 receptors. A further in vitro binding test to clarify the active compound showed that geissoschzine methyl ether (GM), an alkaloid in uncaria hook, a galencial constituent of TJ-54, had a similar potent binding to 5-HT1A and DA2 receptors [25,41,42].

Treatment with TJ-54 at dosages from 2.5 to 7.5 g/day associated with significant amelioration of irritability, including aggression, self-injury, tantrums, lethargy, stereotypy, hyperactivity, and inappropriate speech. In light of research suggesting that a disregulation of DA and 5-HT contributes to maladaptive behavior in PDDs [43], TJ-54’s unique mechanism of action as a partial D2 agonist, 5-HT1A agonist, and 5-HT2A antagonist [25], may prove important for both its effectiveness and tolerability in PDD-NOS and Asperger’s disorder.

The significant reduction on the ABC-I subscale from baseline to end point is noteworthy in that the subjects in this study had higher baseline irritability subscale scores than those in the RUPP Autism Network study of risperidone for irritability in autism [33]. This finding highlights the fact that youths with PDDs other than autism, such as PDD-NOS, often suffer from a significant degree of similar symptomatology [44].

Although highly speculative, these positive changes in socialization may be due to TJ-54’s mechanism of action as a partial 5-HT1A agonist [25]. A putative association has been hypothesized between partial agonism at 5-HT1A receptors and improvements in anxiety and depression, as well as the negative symptoms of schizophrenia [24]. Thus, it is possible that TJ-54 targets these symptoms, thereby potentially resulting in subjects’ increased ability and/or interest in interacting with others. It also may be that by decreasing irritability, the children and adolescents were better able to improve their social functioning over time. Overall, TJ-54 was well tolerated, with no severe or serious adverse effects associated with the drug.

The preliminary results of this study suggest that TJ-54 has the potential to be an effective and well-tolerated treatment for severe irritability in pediatric patients with PDD-NOS, and possibly in Asperger’s disorder. PDD or autism spectrum disorder, range from a severe form, called autism, to a mild form, AD and PDD-NOS. In previous studies almost AD and PDD-NOS children were not mentally retarded (high-functioning). To reduce the bias by heterogeneity of the PDD, in this study, we include mild form PDD, AD and PDD-NOS, and exclude moderate or severe form of PDD, autism.

### Limitations

Given the design characteristics of this trial, the present findings should be taken cautiously. Study limitations include its open design, lack of a control group, and small sample size. In an open-label study, both placebo effects and the role of previously prescribed medication prior to wash out cannot be ruled out as explanations for the observed improvement. Furthermore, regression to the mean is a ubiquitous phenomenon in repeated data that should always be considered as a possible cause of an observed change [45]. However, the rapid improvement in symptoms over several weeks observed here is consistent with a response related to therapeutic dosages of TJ-54. In this study, diagnoses were made by child and adolescent psychiatrist experienced in the diagnosis of PDDs using DSM-IV-TR criteria. However, it will be important to incorporate additional diagnostic instruments into future studies to augment the diagnostic assessment of individuals with subtypes of PDDs, such as the Autism Diagnostic Interview-Revised [46]. Finally, one might consider this sample as not completely representative of the whole population of PDD-NOS and Asperger’s disorder clients because only outpatients not suffering at present from axis I disorders were included, and no subjects had IQ below 70. On the other hand, PDD-NOS and Asperger’s disorder is a heterogeneous condition, and given the present small sample size, treatment efficacy data were better interpreted in a sample that was as homogeneous as possible. In summary, our trial suggests that TJ-54 therapy is a well-tolerated and effective remedy that improves the symptoms of PDD-NOS and Asperger’s disorder. This study has several limitations that could potentially impact the reliability and validity of these findings. Because of its open-label design, bias as well as a placebo effect could be a factor in our findings of improvement in irritability, as well as having provided socialization opportunities to these relatively high functioning individuals every 2 weeks. The number of subjects in this study was relatively small. In addition, the absence of a control group limits the conclusions that can be definitively drawn regarding the safety and tolerability of TJ-54 in this population.

## Conclusions

Although at present there is little evidence of advantages of one antipsychotic over another, the present findings would suggest that TJ-54 is suitable for several symptoms. Present data need to be confirmed in larger, randomized, double-blind trials.

## Abbreviations

TJ-54: Yokukansan; BPSD: Behavioral and psychological symptoms of dementia; BPRS: Brief Psychiatric Rating Scale; HAM-D: Hamilton Rating Scales for Depression; GAF: Global Assessment of Functioning; CGI: Clinical Global Impression Scale; ABC-I: Aberrant Behavior Checklist-Irritability subscale score; PDD-NOS: Pervasive developmental disorder not otherwise specified.

## Competing interests

The authors declare that have no competing interests.

## Authors’ contributions

MT, WR, FM, LK, IM, and JH contributed to the concern and design, acquisition of data, analysis and interpretation of data and drafting of the manuscript and its critical revision for important intellectual content. KK, TK, and IT was involved in the interpretation of data and in the critical drafting and revising of the manuscript for important intellectual content. All authors read and approved the final manuscript.

## Pre-publication history

The pre-publication history for this paper can be accessed here:

http://www.biomedcentral.com/1471-244X/12/215/prepub
